# Seat Belt Injury Diagnosed as Perforation of the 4th Portion of the Duodenum Two Days after a Traffic Accident: A Case Report

**DOI:** 10.70352/scrj.cr.25-0463

**Published:** 2025-10-08

**Authors:** Yuichiro Furutani, Tetsuro Oda, Kei Sugano, Daisuke Fujimori, Takahiro Yoshimura, Koichiro Sawada, Hironori Hayashi, Kaeko Oyama, Masanori Kotake, Takuo Hara, Noriyuki Inaki

**Affiliations:** 1 Department of Surgery, Koseiren Takaoka Hospital, Takaoka, Toyama, Japan; 2 Department of Surgery, Asanogawa General Hospital, Kanazawa, Ishikawa, Japan; 3 Department of Gastrointestinal Surgery/Breast Surgery, Kanazawa University Hospital, Kanazawa, Ishikawa, Japan

**Keywords:** seat belt trauma, duodenal injury, traffic accident, the 4th portion of the duodenum, delayed duodenal perforation

## Abstract

**INTRODUCTION:**

Car seat belts are safety devices that protect passengers during collisions in traffic accidents. However, seat belts can cause abdominal injuries during collisions. Many cases of injury to various intraperitoneal organs caused by seat belts have been reported, but reports of duodenal injury are relatively rare. In addition, diagnosing duodenal injury due to blunt trauma is not easy, and it is not uncommon for cases to be diagnosed long after injury. Here, we report a case in which a patient was diagnosed with perforation of the 4th portion of the duodenum 2 days after injury in a traffic accident.

**CASE PRESENTATION:**

A 67-year-old woman was transported to our hospital after driving a car that collided with a wall. A seat belt mark was observed on her abdomen. A CT scan performed on the day of the injury confirmed a small poorly enhanced area in the duodenum, but no obvious signs of intestinal perforation were found. CT scan also revealed abdominal aortic dissection, a right clavicle fracture, and bilateral rib fractures. Two days later, a CT scan revealed expansion of the poorly enhanced area of the intestinal wall and a small amount of free air in the 4th portion of the duodenum. As duodenal perforation was suspected, emergency surgery was performed. Laparoscopic surgery was performed, and a quarter-circumferential perforation was found in the 4th portion of the duodenum. The perforation was closed with barbed sutures. The patient began oral intake of fluids on POD 7. The abdominal aortic dissection improved with conservative treatment, and the patient was discharged on POD 60 without any postoperative complications.

**CONCLUSIONS:**

We report a case of duodenal injury and abdominal aortic dissection caused by a seat belt during a collision. The reason may be that the seat belt moved toward the upper abdomen during the collision, causing compression of the abdominal organs between the seat belt and the spine. Considering the possibility of delayed duodenal perforation, it is important to perform a CT scan again and select an appropriate treatment according to the situation.

## Abbreviation

US
: ultrasonography

## INTRODUCTION

A seat belt is a safety device that protects the head and chest of a passenger from collisions with interior structures such as the steering wheel and windshield during a traffic accident. However, a seat belt (waist belt) can cause abdominal injury in a car collision. Cases of seat belt injury to various intraperitoneal organs, such as the small intestine, colon, abdominal aorta, mesentery, and duodenum, have been reported (**Table 1**). Among these, reports of duodenal injury caused by a seat belt are relatively rare, and there have been no reported cases of injury to the 4th portion of the duodenum. Furthermore, diagnosing duodenal injury due to blunt trauma is not easy, and it is not uncommon for the diagnosis to be made long after the injury. Here, we report a case in which the injury was diagnosed 2 days after a vehicular collision as the 4th portion of the duodenum, caused by a seat belt, wherein the patient's life was saved. In addition, we examined the mechanism of abdominal injury caused by a seat belt during an accident and in previously reported cases.

**Table 1:** Summary of reports on seat belt-related abdominal injuries (2001–2025)

Year	Author	Age	Sex	Organ injury	Skeletal injury	Surgery date	Surgery detail	Outcome
2001	Voellinger^17)^	21	M	Abdominal aortic transection	None	Day 0	Right hemicolectomy	Survival
				Small intestine injury				
							Resection of small bowel	
				Right mesocolon injury				
2004	Shope^18)^	35	F	Hemorrhagic cholecystitis	None	Day 0	Laparoscopic cholecystectomy	Survival
2011	Iwase^19)^	29	M	Left diaphragm injury	None	Day 0	Suture of the left diaphragmatic	Survival
				Splenic injury				
							Repair of the transverse mesocolon	
				Transverse mesocolon injury				
				Left kidney injury				
2014	Bankar^1)^	26	M	**Duodenum perforation (3rd portion, Grade II)**	None	Day 1	**Suture of the duodenal perforation**	Survival
2015	Mori^20)^	65	F	Gastric wall laceration	None	Day 0	Exploratory laparotomy	Survival
2015	Afifi^21)^	40	M	Left diaphragm injury	Left middle femur fracture	Day 0	Repair of diaphragmatic	Survival
				Mesenteric tear	Right radius fracture		Insertion of left thoracostomy tube	
				Rectal serosa tear	Fibula fracture		Anastomosis of jejunum	
2015	Afifi^21)^	41	F	Small intestine transection	Right distal radius open fracture	Day 0	Repair of diaphragm	Survival
				Mesenteric injury	Rib fracture		Insertion of left thoracostomy tube	
				Left diaphragm rupture	Spinal cord injury			
							Repair of mesenteric tear	
2016	Al-Ozaibi^22)^	24	F	Small intestine transection	Radius and ulna shaft fractures	Day 3	Resection of small bowel	Survival
2016	Al-Ozaibi^22)^	31	M	Mesenteric injury	Right elbow open fracture	Day 1	Right hemicolectomy	Survival
							Resection of small bowel	
2020	Stern^23)^	60	M	Jejunal perforation	Lumbar compression fracture	Day 0	Suture the perforated jejunum	Survival
2023	Ooya^24)^	20s	F	Hepatic subcapsular hematoma	None	No operation	No operation	Survival
2024	Florou^25)^	12	M	Rupture of the abdominal aorta	Right clavicle fracture	Day 0	Endovascular stenting	Survival
				Splenic injury	Bilateral pneumothorax			
				Epidural hematoma	Bilateral pulmonary contusions			
2024	Kim^2)^	15	F	**Duodenum transection (3rd portion, Grade IV)**	Rib fracture	Day 0	**Pancreaticoduodenectomy**	Survival
							Primary portal vein repair	
				Pancreatic injury				
							Right hemicolectomy	
				Retroperitoneal hematoma				
				Left lobe of liver laceration				
				Splenic laceration				
2024	Suzuki^26)^	34	M	Serosa injury of the ascending colon	Right clavicle fracture	Day 0	Right hemicolectomy	Survival
				Mesocolonic hematoma				
							Suture of the perforated ileum	
				Ileal perforation				
2024	De Jesus^27)^	51	M	Aortic occlusion	Lumbar fracture	Day 0	Anastomosis of the small bowel	Death
							Resection of the sigmoid colon	
							Colostomy	
				Sigmoid colon transection				
2025	Serikyaku^3)^	42	F	**Duodenum perforation (2nd portion, Grade II)**	Thoracic vertebrae dislocation/fracture	Day 2	**Suture of the duodenal perforation**	Survival
					Rib fracture			
2025	Our case	67	F	**Duodenum perforation (2nd portion, Grade II)**	Right clavicle fracture	Day 2	**Suture of the duodenal perforation**	Survival
				Abdominal aortic dissection	Rib fracture			

F, female; M, male

## CASE PRESENTATION

**Fig. 1:**
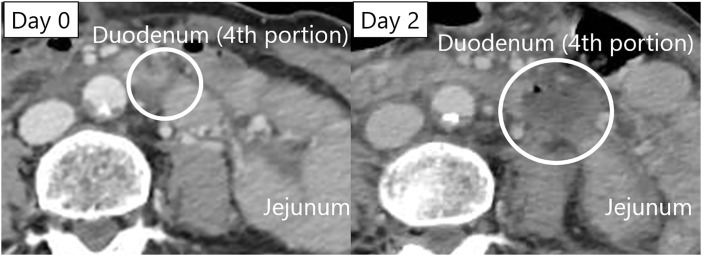
Contrast-enhanced CT findings. Two days after the injury, a CT scan revealed poor contrast (circle) in the intestinal wall of the 4th portion of the duodenum and a very small amount of free air.

A 67-year-old woman who had been driving a car that had collided with a wall was brought to our hospital. Her vital signs were stable, and a seat belt mark was observed on her abdomen. A contrast-enhanced CT scan revealed a very small poorly enhanced area in the 4th portion of the duodenum but no obvious signs of intestinal perforation. Abdominal aortic dissection, right clavicle fracture, and bilateral rib fractures were also found, and conservative treatment was recommended. Two days later, a contrast-enhanced CT scan was performed again to evaluate the abdominal aortic dissection, revealing an expansion of the poorly enhanced area in the 4th portion of the duodenum and a very small amount of free air (**Fig. 1**). As duodenal perforation was suspected, emergency surgery was performed. At that time, although the abdominal pain had not worsened, diffuse abdominal tenderness persisted. The patient’s vital signs were stable, with a blood pressure of 124/92 mmHg and a heart rate of 86 beats/min. Laboratory findings showed leukocytosis (WBC 13400/μL), anemia (Hb 9.0 g/dL), and a platelet count of 198000/μL.

**Fig. 2:**
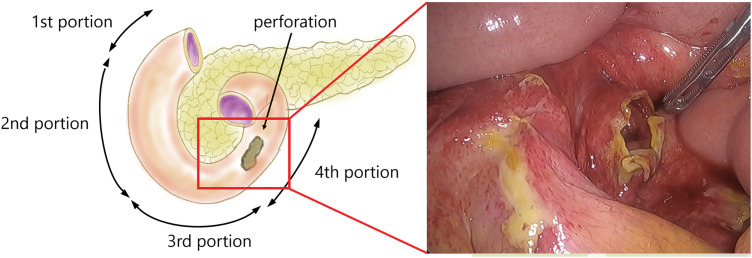
Illustration of the intraoperative findings and surgical findings. A quarter-circumference perforation was observed in the 4th portion of the duodenum.

**Fig. 3:**
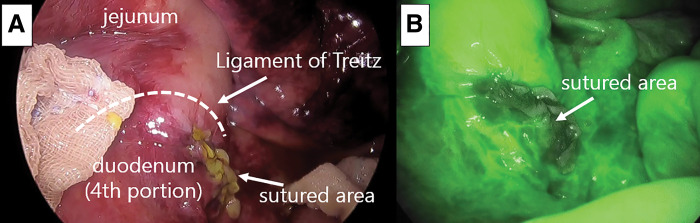
(**A**) Intraoperative image of the sutured area of the duodenal perforation closed with barbed sutures using the over-and-over spiral stitch method. (**B**) ICG fluorescence imaging demonstrating adequate perfusion of the sutured area, confirming no impairment of blood flow at the repair site. ICG, indocyanine green

Laparoscopic surgery was performed. Ports were placed in a configuration commonly used for upper gastrointestinal surgery: a 12-mm port at the umbilicus, a 12-mm port in the right lateral abdomen, and two 5-mm ports in the left lateral abdomen. A small amount of uncontaminated ascites was found in the abdominal cavity, with no evidence of bleeding. A quarter-circumferential perforation was observed in the 4th portion of the duodenum. Since the perforated site was covered by the small intestinal mesentery, no leakage of intestinal fluid was observed (**Fig. 2**). The perforation measured slightly less than 2 cm in length along the longitudinal axis of the duodenum. The color of the perforated duodenal wall was good, and no crushing injury necessitating trimming was observed. The jejunum was retracted to the patient’s right side, which allowed adequate exposure of the 4th portion of the duodenum. At that site, the duodenum was found to be perforated together with the overlying small-intestinal mesentery on its ventral aspect. Primary closure was achieved by continuous suturing with barbed sutures along the longitudinal axis using the over-and-over spiral stitch method (**Fig. 3A**). After suturing, indocyanine green (ICG) fluorescence imaging confirmed adequate perfusion of the sutured area, indicating no impairment of blood flow (**Fig. 3B**). The poorly enhanced area observed on preoperative contrast-enhanced CT was considered to reflect peritoneal fluid collection and inflammatory changes of the intestinal wall due to trauma. The abdominal cavity was washed with physiological saline, a drain was placed, and the surgery was completed. The patient began oral intake of fluids on POD 7 and oral food intake on POD 10. The abdominal aortic dissection improved with conservative treatment, and the patient was discharged on POD 60 without any postoperative complications.

## DISCUSSION

Although there have been some reports of duodenal injury due to blunt trauma, no detailed studies on the location and incidence of duodenal injury have been conducted. A search was conducted on PubMed for the 25-year period from 2001 to 2025 using the keywords “duodenal injury”, “seat belt”, “blunt trauma”, and “traffic injury”. Sixteen cases of abdominal trauma due to seat belts during collisions were reported (**Table 1**). Among these patients, three had duodenal injuries, as in our case.^1–3)^ There are also reports of blunt duodenal injuries other than seatbelt trauma caused by bicycle handlebars^4,5)^ and bullock carts,^6)^ and duodenal injuries can be caused by various triggers.

Although there have been 2 reports of injury to the 4th portion of the duodenum due to blunt trauma,^4,5)^ these injuries were caused by handlebars, and no injury to the 4th portion of the duodenum due to a seat belt was found. Bozkurt B et al.^7)^ reported that the most common site of duodenal injury due to blunt abdominal trauma is the 2nd portion (58%), and it is speculated that injury to the 4th portion of the duodenum alone due to seat belt use is relatively rare.

It is not easy to diagnose intestinal injury due to blunt trauma early using CT scans.^8)^ Bhagvan S et al.^9)^ reported that the predictive sensitivity and specificity of hollow viscus injury using CT scans were 55.33% and 92.06%, respectively, and the positive predictive value and negative predictive value were 61.53% and 89.23%, respectively. In our case, a CT scan on the day of injury revealed a very small poorly enhanced area in the duodenum, but duodenal perforation could not be diagnosed. A CT scan 2 days after injury revealed expansion of the poorly enhanced area and a small amount of free air. This area was later considered to reflect peritoneal fluid collection and inflammatory changes of the intestinal wall due to trauma, consistent with the intraoperative findings. Among the reported cases, there have been occasional cases in which time was required to diagnose intestinal injury, as in our case.^1,3)^ The duodenum is a retroperitoneal organ, so it is covered by fatty tissue and the small intestinal mesentery, and the use of analgesics makes it difficult to judge injury from clinical symptoms, which may also be factors that require substantial time to diagnose. Because delayed diagnosis of duodenal injury leads to increased complications, repeat CT scans and surgery should be considered if delayed injury is suspected.^10)^

**Fig. 4:**
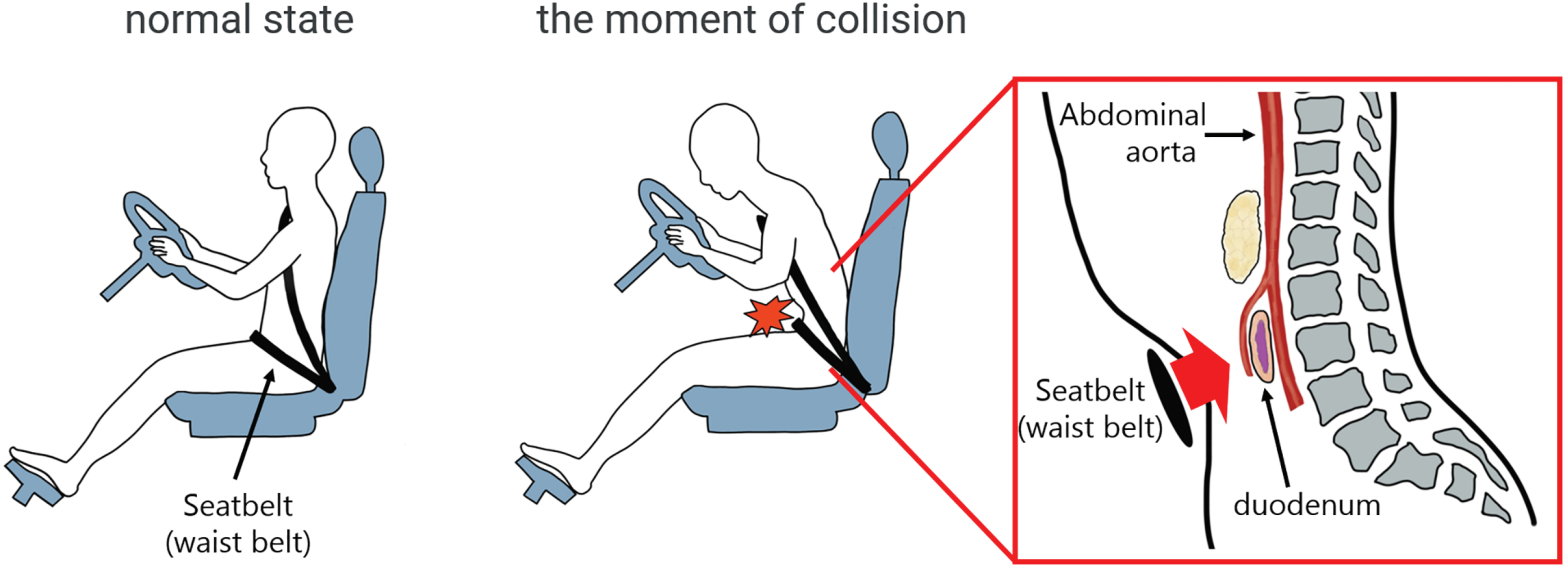
Schematic of the mechanism of duodenal injury caused by a seat belt. The seat belt (waist belt) moves into the upper abdomen, causing the abdominal organs to be compressed between the seat belt and the spine.

Seat belts are safety devices that prevent passengers from moving forward during a collision and from colliding with interior structures of the vehicle. We speculate that the mechanism of abdominal trauma caused by seat belts is as follows (**Fig. 4**). When a car crashes, the body moves forward due to inertial force. The seat belt (waist belt) moves toward the upper abdomen, compressing the abdominal organs between the seat belt and the spine. Cases of duodenal injury often involve other organ injuries.^11)^ As shown in **Table 1**, many cases of seat belt injuries involve not only injuries to a wide range of intraperitoneal organs, such as the small intestine, colon, mesentery, abdominal aorta, and pancreas, but also skeletal injuries. In our case, we observed not only duodenal injury, but also abdominal aortic dissection and skeletal injuries (right clavicle fracture and bilateral rib fractures). It is assumed that the faster the vehicle speed is at the time of collision, the more severe the injury to intraperitoneal organs caused by the seat belt. However, Tominaga et al.^12)^ reported that intraperitoneal organ damage, such as thoracic and liver damage, was observed at speeds of 25 km/h or more, and it is possible that intraperitoneal organ damage may occur due to the seat belt even during collisions at low speeds.

The World Society of Emergency Surgery and American Association for the Surgery of Trauma (WSES-AAST) guidelines provide treatment options for traumatic duodenal injuries according to the severity of the injury.^13)^ In general, Grade I injuries with stable hemodynamics may be managed nonoperatively, but Grade II or higher duodenal injuries require surgical treatment. In our case, the perforation measured slightly less than 2 cm along the longitudinal axis, corresponding to approximately one-quarter of the circumference of the duodenum, which is consistent with Grade II according to the WSES-AAST guidelines. Therefore, primary repair with suture closure was considered appropriate. If the perforation extends beyond half of the intestine, additional surgery or a repair method other than simple suture closure should be considered. Furthermore, in severe cases of Grade III or higher disease, pyloric decompression, biliary diversion, or pancreaticoduodenectomy (Whipple procedure) should be considered depending on the degree of injury.^14)^ There have been reported cases in which emergency pancreaticoduodenectomy was performed and the patient’s life was saved after pancreatic injury due to traffic trauma.^2,15,16)^

To the best of our knowledge, laparoscopic primary repair of a 4th portion duodenal perforation with intraoperative assessment of blood flow using indocyanine green fluorescence has rarely been reported, and this case highlights the feasibility of this minimally invasive approach. Therefore, rapid diagnosis and selection of appropriate treatment for blunt trauma are important. Treatment for blunt abdominal trauma by seat belts varies widely depending on severity, and it is important to select appropriate diagnostic examinations and treatments over time.

## CONCLUSIONS

We reported a case in which a patient presented with duodenal injury and abdominal aortic dissection due to a seat belt during a traffic accident and was diagnosed with duodenal perforation on a CT scan 2 days after the injury. Seat belt trauma can cause extensive damage to abdominal organs and the skeleton, but it can be difficult to diagnose with a CT scan on the day of injury. Considering the possibility of delayed duodenal perforation, it is important to perform a CT scan again and select an appropriate treatment according to the situation.
